# Europium Fluorescent Nanoparticles-Based Multiplex Lateral Flow Immunoassay for Simultaneous Detection of Three Antibiotic Families Residue

**DOI:** 10.3389/fchem.2021.793355

**Published:** 2021-12-20

**Authors:** Yaping Wang, Biao Ma, Miaomiao Liu, Erjing Chen, Ying Xu, Mingzhou Zhang

**Affiliations:** Zhejiang Provincial Key Laboratory of Biometrology and Inspection and Quarantine, China Jiliang University, Hangzhou, China

**Keywords:** sulfonamide antibiotics, tetracycline antibiotics, fluoroquinolone antibiotics, europium nanoparticle fluorescence immunochromatography, multi-residue detection

## Abstract

A fluorescent immunoassay based on europium nanoparticles (EuNPs-FIA) was developed for the simultaneous detection of antibiotic residues, solving the problems of single target detection and low sensitivity of traditional immunoassay methods. In the EuNPs-FIA, EuNPs were used as indictive probes by binding to anti-tetracyclines monoclonal antibodies (anti-TCs mAb), anti-sulphonamides monoclonal antibodies (anti-SAs mAb) and anti-fluoroquinolones monoclonal antibodies (anti-FQs mAb), respectively. Different artificial antigens were assigned to different regions of the nitrocellulose membrane as capture reagents. The EuNPs-FIA allowed for the simultaneous detection of three classes of antibiotics (tetracyclines, fluoroquinolones and sulphonamides) within 15 min. It enabled both the qualitative determination with the naked eye under UV light and the quantitative detection of target antibiotics by scanning the fluorescence intensity of the detection probes on the corresponding detection lines. For qualitative analysis, the cut-off values for tetracyclines (TCs), fluoroquinolones (FQs) and sulphonamides (SAs) were 3.2 ng/ml, 2.4 ng/ml and 4.0 ng/ml, respectively, which were much lower than the maximum residue limit in food. For quantitative analysis, these ranged from 0.06 to 6.85 ng/ml for TCs, 0.03–5.14 ng/ml for FQs, and 0.04–4.40 ng/ml for SAs. The linear correlation coefficients were higher than 0.97. The mean spiked recoveries ranged from 92.1 to 106.2% with relative standard deviations less than 8.75%. Among them, the three monoclonal antibodies could recognize four types of TCs, seven types of FQs and 13 types of SAs, respectively, and the detection range could cover 24 antibiotic residues with different structural formulations. The results of the detection of antibiotic residues in real samples using this method were highly correlated with those of high performance liquid chromatography (*R* ^2^ > 0.98). The accuracy and precision of the EuNPs**-**FIA also met the requirements for quantitative analysis. These results suggested that this multiplex immunoassay method was a promising method for rapid screening of three families of antibiotic residues.

## Introduction

Since the discovery of penicillin in 1929, antibiotics were frequently used as clinical agents to prevent and treat bacterial infections in human and veterinary medicine (Kong et al., 2020). Effective antibiotics were the cornerstone of modern medicine supporting organ transplants, surgical prophylaxis, protecting newborns from septic infections and avoiding infections during cancer chemotherapy. Unfortunately, the excessive and indiscriminate use of antibiotics in interconnected ecosystems around the world had led to a dramatic rise in drug resistance. This concern had been listed by the World Health Organisation as one of the top ten public health threats (Anita et al., 2021). A number of veterinary antibiotics, including tetracyclines (TCs), fluoroquinolones (FQs) sulphonamides (SAs) and macrolides (MLs), were used to improve feed efficiency and promote growth rates. Approximately 30–90% of consumed antibiotics were excreted in urine and faeces as parent compounds and/or metabolites. In addition, 70–80% of the antibiotics used in animal feeding also end up in food due to incomplete removal of antibiotics (Ben et al., 2019). Prolonged exposure of microorganisms to such conditions could transform them into “superbugs”. This places a heavy burden on the global economy. It is estimated that by 2050 some 4.44 million people would be affected by infections that would not receive effective treatment (Chen Y. et al., 2020). The overuse of antibiotics in interconnected ecosystems had led to their presence in a variety of environmental matrices, even in the food chain, which had posed a growing threat to public health safety. For example, antibiotics could modulate human mood and increase the risk of depression (mental illness) by altering the gut microbiota and brain-gut axis (Konstantinidis et al., 2020). It may also induce a range of genetic effects in humans, such as carcinogenic or allergic reactions (Li et al., 2020).

SAs, FQs, and TCs were common food contaminants that were often left in foods such as eggs, milk, chicken and honey (Zou et al., 2019), posing a concerted threat to human health. Excess antibiotics accumulate in the body through food, causing dysbiosis in the human intestinal flora, leading to increased bacterial resistance even cancer in various tissues and organs (Hoffmann et al., 2016). Many countries and government organisations had set maximum residue limits (MRLs) for most antibiotics in different animal foods (Yong et al., 2018). For example, the Ministry of Agriculture of the People’s Republic of China has announced that the MRL for SAs, TCs, and FQs in milk were 25, 100, and 100 ng/ml (GB31650-2019, 2019). Therefore, it was particularly important to test for residues of antibiotics in food. As the number of controlled compounds in medical diagnostics increases (Rashid et al., 2020), food safety and ecological monitoring due to the choice of multiple antibiotics mixed to achieve the desired effect when faced with disease treatment and prevention led to the need for multiple testing systems to ensure public environmental safety (Yi et al., 2016). The increase in health risk factors had placed new demands on analytical methods for medical diagnostics, environmental monitoring and food quality control. In these areas, it was important to be able to monitor the levels of several antibiotics in the same sample simultaneously and to obtain test results in a short time with minimal labour (Jin et al., 2020). However, the application of europium fluorescent labelling for the simultaneous detection of multiple antibiotics had been rarely reported. In recent years, various methods had been developed for the detection of ciprofloxacin (El Kojok et al., 2020), tetracycline (Xu et al., 2020) and sulfadimethoxine (Yan et al., 2017) in food, but the detection targets were single and the sensitivity was not satisfactory (Chen Y. et al., 2020). In a global study (Amy et al., 2020), the presence of three and more different species of antibiotic residues could be detected simultaneously in different environmental matrices in 47 countries worldwide, with TCs, SAs, and FQs accounting for a large proportion of these residues (Chen H. et al., 2020). Therefore, the detection of a single type of antibiotic residue was no longer sufficient to ensure the safety of the public living environment.

With increasing concern for environmental quality and public health (Zhang et al., 2020) real-time monitoring of antibiotic residues in the aquatic environment is essential (Yang et al., 2020). There were many methods for screening antibiotic residues (Zhen et al., 2011), including chromatography, microbiological assay and immunoassay (Wang et al., 2019). Although the microbiological method was simple and convenient, the specificity was relatively low, and it required time-consuming sample processing steps (Wang et al., 2019). Chromatographic methods, such as high performance liquid chromatography (HPLC) (Soh et al., 2020), capillary electrophoresis (CE) (Asha et al., 2020), mass spectrometry (MS) (Abafe et al., 2020) are characterized by high sensitivity and reliability. However, special laboratories with expensive equipment and high-quality personnel were demanded. Obviously, the application of these methods in on-site detection of antibiotic residues in food was limited (Suo et al., 2019).

In addition, there are many immunoassays in use, such as radioimmunoassay (RIA) (Zhou et al., 2014), flow injection chemiluminescence with immunoassay (FICLIA) (Yuan et al., 2017), colloidal gold immunoassay (GIA) and enzyme-linked immunoassay (ELISA) (Song et al., 2018). Among these, ELISA was used as a validated immunoassay for screening a large number of samples. However, enzyme-linked immunosorbent assay still needs complicated and precise laboratory operations, such as multiple incubation, washing and sample pretreatment steps, which hinders its application in society and market (Lin et al., 2019). In resource-poor areas, lateral flow immunoassay strips (De et al., 2020), were more feasible than ELISA. It had been widely used for screening veterinary drug residues in food because of their unique advantages of rapidity, simplicity, stability and portability as an alternative choice to ELISA in essential sites (Chen H. et al., 2021) and had been widely used for screening veterinary drug residues in food (Ling et al., 2019). Among them, the commonly used colloidal gold immunoassay had limited its wide application in production and development due to matrix effect and low sensitivity (Liang et al., 2019; Marjan et al., 2019). In order to improve the sensitivity and specificity of the method for quantitative and multi-drug residue detection, some new immune-chromatographic detection methods have gradually highlighted their unique advantages in residue detection (Ben et al., 2019).

As an alternative to colloidal gold, fluorescent markers such as quantum dots, fluorescent nanosilica, fluorescent beads and lanthanides have been widely used in immunochromatographic methods (Wei et al., 2019). As the excitation and emission wavelengths of most fluorescent markers are in the ultraviolet-visible (UV-vis) wavelength range, membrane supports, biological components etc. generate high fluorescence background interference due to light scattering and autofluorescence (Castillo-García et al., 2015). The advantage of the strong signal intensity contributed by these fluorescent markers is therefore diminished by their high fluorescence background (Ye et al., 2020). The lanthanides (Wang et al., 2020), europium (Eu) (II), terbium (Tb) (II), samarium (Sm) (II) and dysprosium (Dy) (III), were of interest because of their long fluorescence lifetimes and large Stokes shifts (Chen H. et al., 2020), which help to reduce the interference of fluorescence background signals and improve sensitivity. It can be seen that lanthanide-europium was already used for the detection of streptomycin in milk (Anastasiya et al., 2020), however, the detection was limited by the single target.

In this study, we introduced a europium nanoparticle-based fluorescent immunoassay technique (EuNPs-FIA) for the simultaneous determination of tetracyclines, fluoroquinolones, and sulphonamides antibiotics in food. The problem of single detection target and low sensitivity was solved. Three population-specific monoclonal antibodies were developed to increase the number of tests. The three monoclonal antibodies could recognize four types of TCs, seven types of FQs and 13 types of SAs, respectively, and the detection range could cover 24 antibiotic residues with different structural formulations. Together with the advantage that the lateral flow immunoassay strips can be easily manipulated, it makes the whole assay system of great potential for quality control and detection of drug residues.

## Materials and Methods

### Reagents

Tetracycline (TC), Chlortetracycline (CTC), Oxytetracycline (OTC), Doxycycline (DOX), Enrofloxacin (ENR), Ciprofloxacin (CIP), Ofloxacin (OFL), Lomefloxacin (LOM), Dalflo-xacin (DAL), Norfloxacin (NOR), Difluoxacin (DIF), Sulfamethoxypyridazine (STD), Sulfisoxazole (SXZ), Sulfamethoxypyrimidine (SFM), Sulfathiazole (SFZ), Sulfaquinoxaline (SFX), Sulfapyridine (SFD), Sulfamethoxazole (SMA), Sulfaclozine (SFO), Sulfadimethoxypyrimidine (SMD), Sulfamethoxypyrimidine (STM), Sulfamethoxypyrimidine (SPM), Sulfadimethoxypyrimidine (SMM) and Sulfadiazine (SMZ) were purchased from the National Institute of Metrology, P. R. China (Beijing, China). 1-(3-dimethylaminopropyl)-3-ethylcarbodiimide (EDC), N-hydroxysuccinimide (NHS), 2-(N-Morpholino) ethanesulfonic acid (MES) were obtained from Sigma-Aldrich (St. Louis, MO, United States). Bovine serum albumin (BSA), human serum albumin (HAS), were purchased from Sino-American Biotechnology (Luoyang, Henan, China). Enzyme immunoassay-grade horseradish peroxidase-labeled goat anti-mouse immunoglobulin, freund’s complete and incomplete adjuvant was purchased from Aladdin Industrial Corporation (Shanghai, China). Nitrocellulose (NC) membrane, sample pad, conjugate pad and absorbent pad were obtained from Dean Biotechnology Co, Ltd (Hangzhou, Zhejiang, China).

Phosphate buffer solution (PBS, 0.01 M, pH 7.4) was prepared by weighing 0.4 g NaCl, 3.1 g disodium hydrogen phosphate and 0.5 g dipotassium hydrogen phosphate dissolved in ultrapure water and fixed to a final volume of 2 L, followed by adjustment of pH 7.4 with sodium hydroxide. Borate buffer solution (BBS, 0.05 M, pH 8.2) was prepared by weighing 1.68 g boric acid and 1.34 g NaB_4_O_7-_10H_2_O was dissolved in ultrapure water, 100 μl of 1% Tween-20 solution was added and the final volume was fixed to a final volume of 2 L to prepare a borate buffer solution containing 0.05% (v/v) Tween-20. Carbonate buffer solution (CBS, 0.1 M, pH 9.6), 3.18 g Na_2_CO_3_ and 5.88 g sodium bicarbonate were weighed, dissolved in ultrapure water and allowed to reach a final volume of 2 L. MES buffer solution (0.05 M, pH 6.5), 9.76 g MES was weighed, dissolved in ultrapure water and allowed to reach a final volume of 1 L, then adjusted with NaOH (pH 6.5).

### Instruments

The role of the Hitachi F-4500 fluorescence spectrometer system (Hitachi, Tokyo, Japan) was used to read and record the fluorescence pattern. An XYZ3000 dispensing platform and a CM2000 guillotine cutter (BioDot, Irvine, CA, United States) was used to prepare and cut test strips. The purpose of the FIC-S2011-B14 fluorescent strip reader (Suzhou, Jiangsu, China) was used to read and record the results of the fluorometric test strip assay. Agilent 1,100 high performance liquid chromatography system (Agilent Tech, Santa Clara, CA, United States) was used to monitor the results of validated fluorescent test strips.

### Synthesis of Artificial Antigens and Immunogens

Based on previous work in our laboratory (Gong et al., 2014), the parent nucleus structures of sulphonamide antibiotics, tetracycline antibiotics and fluoroquinolone antibiotics were selected as antigens to obtain immunogenicity by binding to carrier proteins. TCs and SAs were synthesised as artificial antigens using a diazotization method, whereby the amino group on the parent nucleus structure was activated using sodium nitrite, and the antigens were then bound to BSA and HSA to prepare immunogen. For FQs, the carboxyl group on the parent nucleus was activated using EDC/NHS and the antigen was bound to BSA and HSA to produce the immunogen and artificial antigen. SDS-PAGE electrophoresis and UV-Vis were used to identify the couples and to evaluate the coupling process.

### Preparation of Monoclonal Antibody

Bal/c female mice were selected as immune animals to produce antibodies. In short, each mouse was subcutaneously injected with 100 μg of immunogen HSA-SAs, HSA-TCs and HSA-FQs emulsified with freund’s complete adjuvant, respectively, once a week. After 4 weeks, the immunization was strengthened, each mouse was subcutaneously injected with 50 μg of immunogen emulsified with freund’s incomplete adjuvant. After three times of immunization, the mouse serum was analyzed and identified by ELISA using homologous and heterologous coated antigens. When the serum titer reached the highest, the mouse spleen with the lowest serum titer and lowest half-maximal inhibitory concentration (IC_50_) value was taken out, and the spleen cells were fused with Sp2/0 myeloma cells. 96 h before cell fusion, the last spleen shock was performed. After cell fusion, hybridoma cell lines secreting anti-SAs, TCs and FQs monoclonal antibody was screened out by selective medium respectively, and the three cell lines were subcloned three times respectively, and the results of each subclone were identified by ELISA (Gong et al., 2014). Screening the best cell line, injecting the best cell line into the abdominal cavity of mice perfused with paraffin to produce ascites, finally purifying the ascites by saturated ammonium sulfate method to obtain anti-SAs, anti-TCs and anti-FQs monoclonal antibody respectively, measuring the antibody concentration by UV-Vis, and storing at −20°C.

### Preparation of EuNPs-mAb Probes

Add 3 mg carboxylated europium and 45 μl EDC (10 mg/ml) to 1,200 μL MES solution (0.05 M, pH 6.5), and activated by incubation at room temperature on a shaker at 200 rpm/min for 30 min. The activated product solution was centrifuged at 15,000 rpm for 20 min. After separating additional EDC, the precipitate was redissolved in 500 μl BBS (0.05 M, pH 8.0) solution. The solution was divided into fifteen aliquots and 1 ml of anti-FQs mAb at concentrations of 1, 2.5, 5, 15 and 20 μg/ml, 1 ml of anti-TCs mAb at concentrations of 2.5, 5, 10, 20 and 30 μg/ml and 1 ml of anti-SAs mAb at concentrations of 10, 15, 20, 25 and 30 μg/ml were added, respectively and incubated with shaking at room temperature for 2 h. At the end of the reaction, 55 μl 10% BSA (w/v) was added to continue shaking incubation for 2 h, respectively. The excess antibody and BSA were separated by centrifugation three times at 15,000 rpm/min, 10 min. At last, the precipitate was dissolved in 500 μl BBS (0.05 M, pH 8.0) containing 0.1% BSA (w/v), and stored at 4°C until further use.

### Assembly of Immunochromatographic System

The immunochromatographic test strip consists of four parts, namely, an absorption pad, a binding pad, a sample pad and a nitrocellulose membrane, which are sequentially assembled on a backing card, and all components are assembled with the back plate overlapped by 2 mm. Sample pad was soaked in PBS buffer solution (0.01 M, pH 7.4) containing 1% BSA (w/v) and 0.05% Tween-20 (v/v), then dried overnight in a drying oven at 37 °C and stored in a sealed bag with desiccant for later use. The nitrocellulose membrane was soaked in PBS buffer solution (0.01 M, pH 7.4), dried at 37°C for 12 h, and then dried for storage. SAs-BSA, FQs-BSA, TCs-BSA and goat anti-mouse IgG (GAM-IgG) were sprayed on the T and C lines of the nitrocellulose membrane respectively. The labelled monoclonal antibodies against TCs, FQs and SAs were diluted in BBS buffer (0.05 M, pH 8.2, containing 8% (w/v) sucrose and 1% (w/v) BSA) respectively. They are sprayed separately on the bonding pad. The scratched nitrocellulose membrane were dried overnight at 37°C. After the above components are processed, they can be assembled into a complete immunochromatographic system, which is cut into 2.5 mm test strips and dried and stored at room temperature for later use.

### Optimisation of Parameters for Single-Component Immunochromatography Systems

Based on the influence of various factors on the fluorescence intensity and sensitivity of the test results, several key parameters were optimized systematically, including the concentration of EuNPs labeled monoclonal antibody, the probe addition volume, artificial antigen encapsulation concentration and GAM-IgG encapsulation concentration, so as to obtain higher sensitivity and fluorescence signal intensity.

The membrane concentration of SAs-BSA and goat anti-mouse horseradish peroxidase was optimized from 0, 0.2, 0.4, 0.6, 0.8, 1.0 mg/ml and 0, 0.5, 1.0, 1.0, 2.0, 2.5 mg/ml respectively.

After optimizing the above variable conditions, the fluorescent probes with the concentrations of 2, 4, 6, and 8 ng/ml were dripped into the test strips, and the optimal amount of probes was screened by detecting the fluorescence intensity of C line and T line.

Similar parameter optimisation was carried out for single test strips of FQs and SAs.

### Optimization of the Multi-Immunochromatographic System

Based on the optimised parameters of the single immunoassay system, The parameters of the triple fluorescence immunochromatography system, such as artificial antigen scribe position, encapsulation concentration and GAM-IgG encapsulation concentration, were optimised by orthogonal test L9 (3)^3^ analysis.

At 5, 10, 15, 20, 25, 30, 35, and 40 min after the start of the reaction, the fluorescence intensity of the C-line and T-line of the test strip was detected, and the optimal reaction time was screened by comparison.

### Specificity and Sensitivity Assessment

The cross-reactivity (CR) and the limit of detection (LOD) were used as quantitative parameters to measure the specificity and sensitivity of this immunochromatographic system. A calibration curve was constructed by plotting the ratio of the fluorescence intensity of spiked and blank samples (B/B_0_) against the logarithmic concentration of the analyte.

Under optimised conditions, fixed concentrations of mixed standard solutions were prepared by adding each type of antibiotic drug to the buffer solution, and the standard curve was established by testing a series of these solutions and reading the values through a fluorescence reader.

The standard curve was obtained by plotting the logarithm of the optical density or fluorescence intensity (Y) versus the concentration of the analyte (X) and fitted to a linear equation. The linear range for this analysis was set at the concentration that results in 10–80% inhibition.

Standard solutions of 13 types of SAs were diluted to a final concentration of 500 ng/ml and tested separately against a single sulphonamide immunochromatographic system. Standard solutions of seven types of FQs were diluted to a final concentration of 500 ng/ml and tested separately on a single fluoroquinolone immunochromatographic system. Standard solutions of four types of TCs were diluted to a final concentration of 500 ng/ml and tested separately on a single tetracycline immunochromatographic system. The specificity of the triple immunochromatographic system was assessed by testing each analyte individually to exclude false positive results, using the respective mixtures as reference analytes.

### Spiked Recovery Tests and Analysis of Actual Samples

To assess the reliability of the developed test strips, spiked samples in the concentration range of 0.1–2.4 ng/ml were prepared by adding three representative antibiotic drug standards to a blank negative aqueous solution under optimised conditions and analysed for identification using the EuNPs-FIA developed in the above text.

In order to verify the practical applicability of EuNPs-FIA, four types of actual samples, including honey, milk, chicken and eggs, were selected for analysis. The samples were pre-treated with reference to other methods (Anastasiya et al., 2020).

Chopped chicken was homogenized at 10,000 r/min for 1 min 1.0 g of homogenate was weighed into a 15 ml centrifuge tube and 5 ml of acetonitrile was added. The mixture was vortexed and shaken for 5 min and centrifuged at 4,000 r/min for 5 min at room temperature. 1.5 ml of supernatant was transferred to a 5 ml centrifuge tube and dried under nitrogen at 55°C. Then 1 ml of hexane was added to the residue. Vortex for 30 s and then add 1 ml of PBS. The lower liquid phase was collected by centrifugation and filtered through a 0.22 µm filter for detection. A solution of 950 μl could be collected and 40 μl was the loading volume for 1strip. The pre-treatment process was similar for chicken and honey. Weigh 1.0 g of honey into a 15 ml centrifuge tube. Add 5 ml of acetonitrile. Vortex and shake the mixture for 5 min. The next steps were the same as for the chicken. The pre-treatment process for the eggs was slightly different. After shelling the eggs, homogenise at 500 r/min for 20 s to mix the whites and yolks. 1.0 g of homogenate was accurately weighed and placed in a centrifuge tube. Then, 2 ml of PBS was added and the homogenate was shaken gently by hand for 20 min. After centrifugation at 4,000 r/min for 10 min at room temperature, the supernatant was collected and passed through a 0.22 μm filter for detection. A solution of 1.95 ml can be collected and 40 μl was the loading volume for 1 strip. The pre-treatment process for milk samples was the simplest. The milk sample was diluted five times directly using PBS solution for the assay. 40 μl was the loading volume for 1 strip.

All actual samples were analyzed and confirmed by high performance liquid chromatography (HPLC). The detection of that actual sample were achieved using 0.08% acetic acid in Milli-Q water, methanol, and acetonitrile with a gradient elution and a Zorbax Eclipse XDB C18 chromatographic column. The flow rate was 0.6 ml/min, and the injection volume was 40 µl. The column temperature was 25°C.

### Data Analysis

The peaks of the fluorescence spectra required in the text were analysed using Origin 9 software (Origin Lab, United States). Graphs such as standard curves were plotted using Microsoft Excel software (Microsoft Corporation, United States) for analysis. Schematics of the test strips were drawn using Photoshop software (Adobe Systems, United States).

## Results and Discussion

### Detection Process of Immunochromatographic System

**FIGURE 1:**
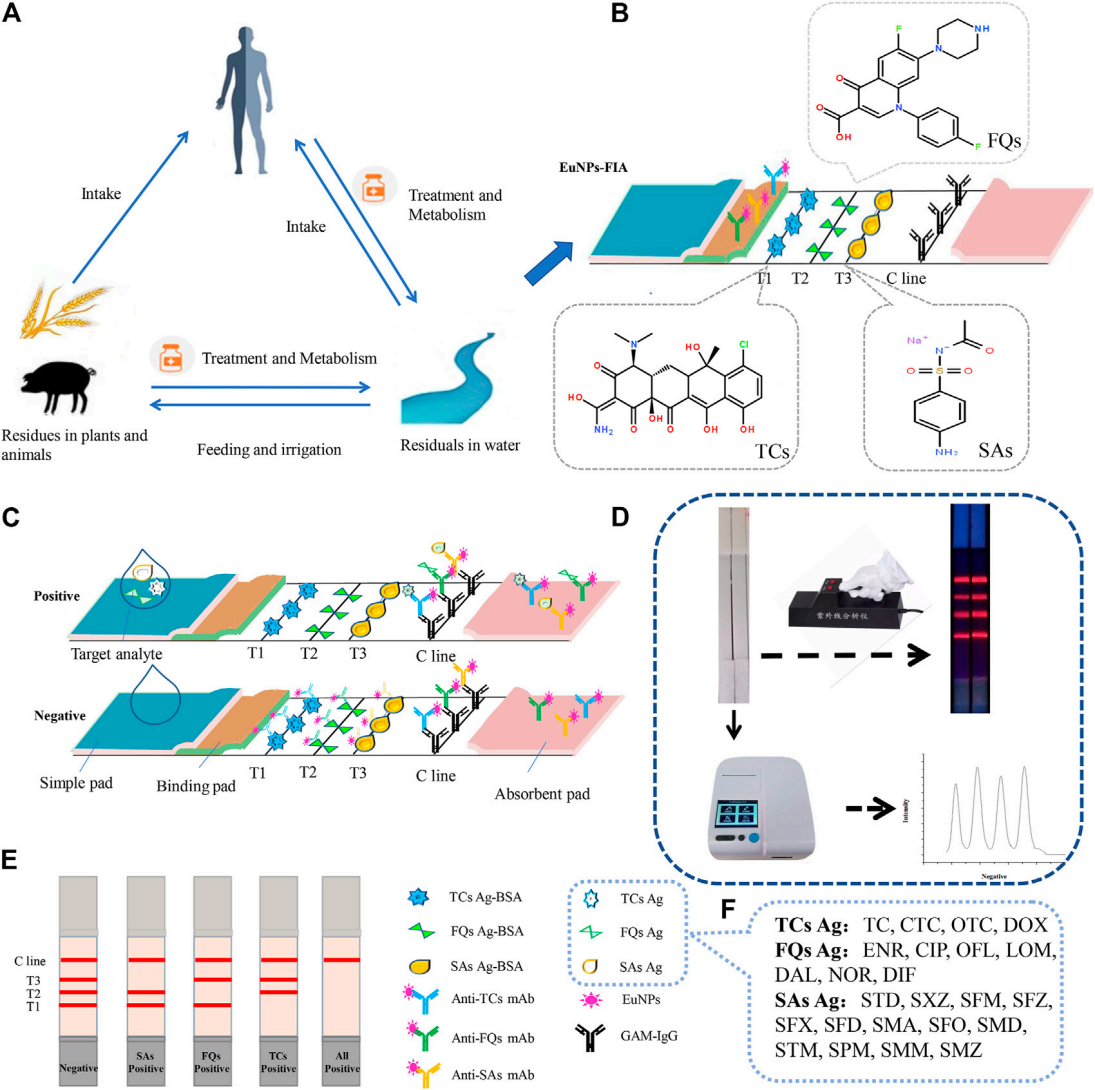
The EuNPs-FIA detection principle. **(A)** The cycle of antibiotics between man and nature. **(B)** Principle of assemble EuNPs-FIA test strips. **(C)** Test results of the EuNPs-FIA reader test results. **(D)** The actual testing process and test results of the EuNPs-FIA reader test results. **(E)** Diagram of the test results. **(F)** Number of detectable target species.

The detection principle of immunochromatography system was based on the competitive reaction theory (Gong et al., 2014), the principle is based on the competition between target detectors contained in the detection sample and artificial antigen conjugates fixed on nitrocellulose membrane, so as to bind to the monoclonal antibody labeled by EuNPs (Figure 1). Three different antigens were coated on nitrocellulose membranes at different locations to act as capture reagents. Three different monoclonal antibodies labelled with EuNPs were immobilised on the binding pad as detection probes. Drop the sample solution mixed with target analyte onto the sample pad. Under the capillary force, the solution flows to the other side. When flowing through the T line, a competitive reaction occurs. When the monoclonal antibodies labelled by EuNPs bind to the target analyte in the sample, would cross over to the artificial antigen on the T-line and instead binding to and accumulating goat anti-mouse IgG on the C-line (Figures 1A,B). On the contrary, when the sample solution does not contain the target analyte, the monoclonal antibody labeled by EuNPs reacted with artificial antigen on T-line and goat anti-mouse IgG on C-line (Figure 1C). The fluorescence intensity on the test strip is read and stored by portable reader. If the test procedure is performed correctly, the control line will always be visualised. For qualitative analysis, the fluorescence image was visually inspected to determine the result, while for quantitative analysis, a standard curve is constructed by plotting the ratio between the fluorescence intensity (B/B_0_) of the spiked and blank samples against the log concentration of the antibiotic residue. The fluorescence intensities of the unknown samples were then brought into the respective calibration curves for calculation to determine the analyte concentrations (Figure 1D-F).

### Evaluation of Artificial Antigens and Monoclonal Antibodies

In order to obtain population-specific monoclonal antibodies that were effective against each antibiotic family, the synthesis of semi-antigens was essential (Lin et al., 2010).

The fluoroquinolone antibiotics were chosen to introduce a fluorine atom in the 6 position and a structure with a basic piperazine substituent replaced at the 7 position in pirimicarbazide as the parent nucleus structure, which was linked to the carrier proteins bovine serum albumin and HSA using the carbodiimide method to prepare the artificial antigen (Supplementary Figure S1).

The recognition of members of the sulfonamides family should be based on the common aminobenzensulfonylamino moiety structure. Sulphonamide antibiotics have a similar parent nucleus structure and different R groups. The R groups can be broadly classified into two categories:five-membered heterocycles and six-membered heterocycles. Sulfadimethoxine contains only the normal aminobenzenesulfonamide structure without any R groups. Therefore, it was chosen as the parent structure of the sulphonamide antibiotic and the artificial antigen was prepared by linking it to the carrier proteins bovine serum albumin and HSA using the N-hydroxysuccinimide activated ester method (Supplementary Figure S2).

The common structure of tetracycline antibiotics was the tetraphenyl tetracyclic skeleton, all containing phenolic hydroxyl, enol hydrocarbon and dimethylamine groups. This structure was chosen as the parent nucleus structure of tetracycline antibiotics and the artificial antigen was prepared by linking it to the carrier proteins bovine serum albumin and HSA using the N-hydroxysuccinimide activated ester method (Supplementary Figure S3).

The SDS-PAGE identification results indicated successful artificial antigen coupling (Supplementary Figure S1–S3).

After cell fusion, the immunised mice produced ascites. The antibodies purified from ascites were identified and evaluated by enzyme-linked immunosorbent chromatography, and the respective antibody with the highest activity was selected (Supplementary Figure S4). Cross-reactivity tests between these three antibodies and other antibiotics of the same class showed good selectivity (Supplementary Table S1–S3). SAs-mAb, FQs-mAb and TCs-mAb could identify 13 classes of SAs, seven classes of FQs and four classes of TCs respectively, enabling the simultaneous detection of the same class of antibiotics on the same T-line, greatly increasing the number of detectable targets by immunochromatographic techniques.

### Optimisation of EuNPs-Antibody Conjugates

**FIGURE 2:**
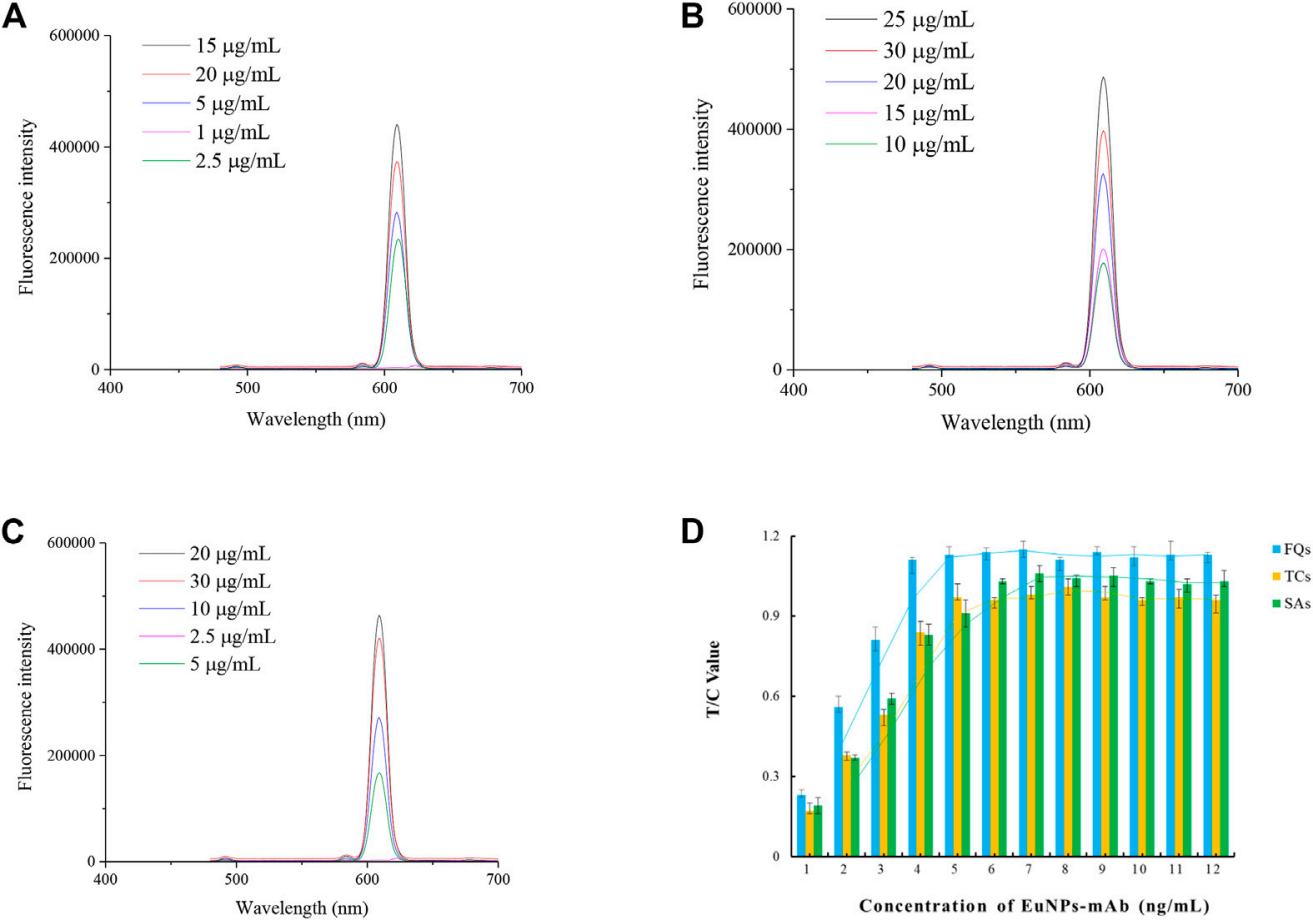
Optimisation of EuNPs-antibody conjugates. **(A)** Effect of the amount of FQs-mAb labelling on the detection capability of the probe. **(B)** Effect of the amount of SAs-mAb labelling on the detection capability of the probe. **(C)** Effect of the amount of TCs-mAb labelling on the detection capability of the probe. **(D)** Effect of probe incorporation on the detection capacity of single-EuNPs-FIA.

The optimisation of the antibody labelling amounts showed that the optimal labelling concentrations for the anti-FQs monoclonal antibody, anti-SAs monoclonal antibody and anti-TCs monoclonal antibody were 15, 25 and 20 μg/ml (Figures 2A–C).

The results of the optimization of fluorescent probe incorporation in the single immunochromatographic system showed that the optimal incorporation amounts of SAs-mAb-EuNPs, FQs-mAb-EuNPs and TCs-mAb-EuNPs were 6, 4 and 5 ng/ml, respectively, which can be used as a reference for the optimization of the parameters of the subsequent triple immunochromatographic system (Figure 2D).

Antibodies and labelling materials were the basis of immunological detection methods (Na et al., 2020). Lanthanide, europium effectively reduces non-specific interference such as background signals, covalently couples to monoclonal antibodies by the active ester method to form detection probes that effectively indicate antibiotic residues in samples and retain satisfactory sensitivity.

### Optimisation of Parameters for Single-Component Immunochromatography Systems

**FIGURE 3:**
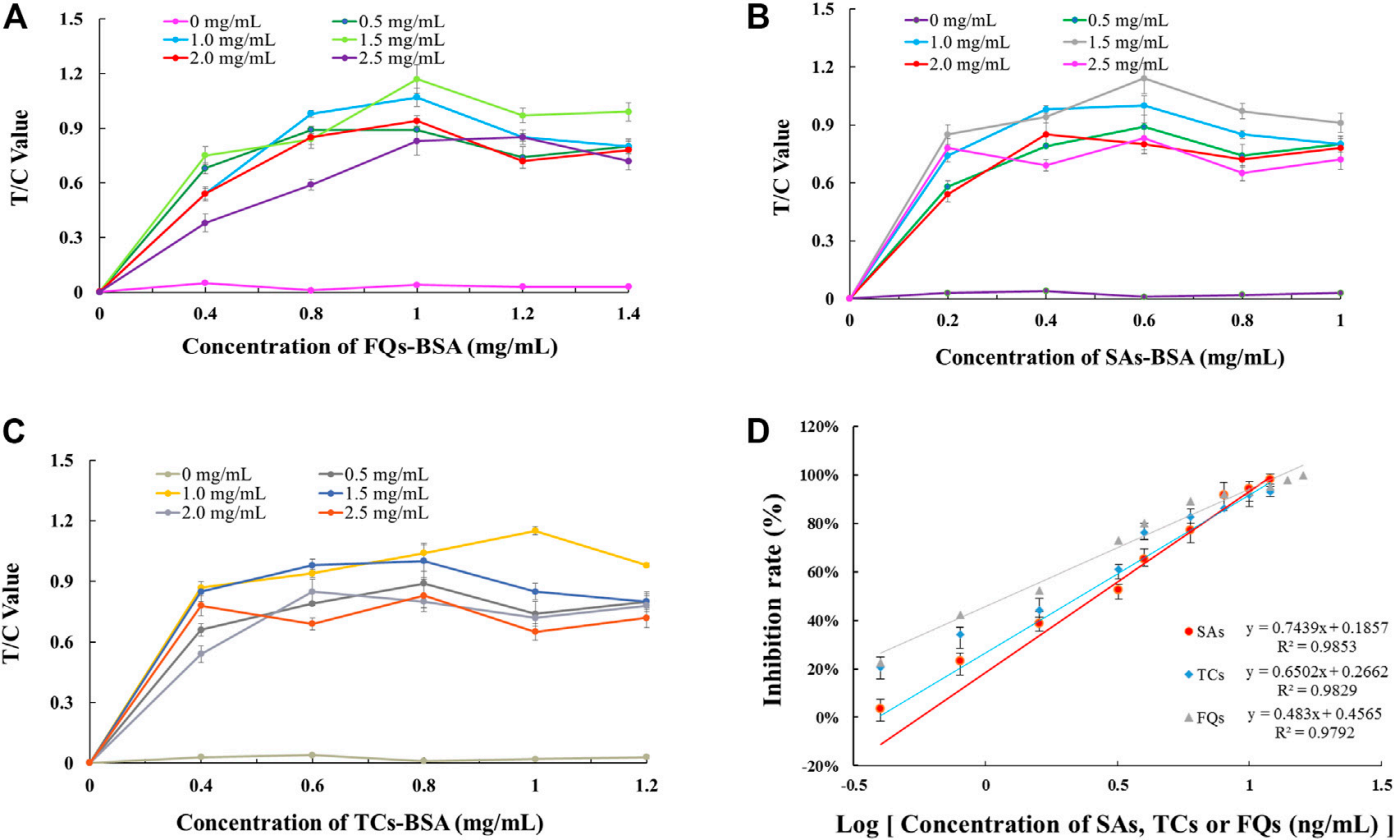
Parameter optimisation of single-EuNPs-FIA. **(A)** Effect of encapsulation concentrations of FQs-BSA and GAM-IgG on the detection capacity of single-EuNPs-FIA. **(B)** Effect of encapsulation concentrations of SAs-BSA and GAM-IgG on the detection capacity of single-EuNPs-FIA. **(C)** Effect of encapsulation concentrations of TCs-BSA and GAM-IgG on the detection capacity of single-EuNPs-FIA. **(D)** Three standard curves for single-EuNPs-FIA.

By optimising the concentrations of artificial antigen and GAM-IgG encapsulated in the T and C line positions on the NC membrane, it was concluded that 1.0 mg/ml of FQs-BSA and 1.5 mg/ml of GAM-IgG were used as the optimal amounts for the chromatographic system for single detection of FQs (Figure 3A). For the chromatographic system for single detection of SAs, 0.6 mg/ml of SAs-BSA and 1.5 mg/ml of GAM-IgG was used as the optimal amount for this system (Figure 3B). For chromatographic systems with single detection of TCs, 1.0 mg/ml of TCs-BSA and 1.0 mg/ml of GAM-IgG was used as the optimal amount for this system (Figure 3C). The above assay results may provide a reference for subsequent optimization of the triple immunochromatographic system parameters. A standard curve was created by reading single test strips using an EuNPs fluorescent strip reader under optimised conditions to assess the detection sensitivity of the single test strip (Figure 3D).

The advantage of the single immunochromatographic technique was that only one specific artificial antigen was encapsulated on the T-line, eliminating the need to consider the reaction between the sample and other specific substances when the liquid sample under test comes into contact with the T-line. Under optimised conditions, single-EuNPs-FIA could simultaneously detect 13 types of SAs, seven types of FQs and four types of TCs respectively, determining its potential as a competitive tool for primary mass screening in comparison to other analytical methods. However, Europium has been applied to the detection of streptomycin in milk, however, the detection was limited by a single target (Luo et al., 2020). The increase in the number of controlled compounds in medical diagnostics and ecological assays makes a single detection system inadequate for the detection needs (Supplementary Table S3).

### Optimization of the Multi-Immunochromatographic System

As the solution moves along the test strip, the concentration and interaction of the reactants in the solution changes as its initial volume is diluted (Chen et al., 2014). This affected not only the chemical equilibrium in the solution but also the binding efficiency of the test probe when it reached the T-line, so that not only the limit of detection but also the intensity of fluorescence development changed significantly in the multiplex immunochromatography technique (Roberta et al., 2014). For multiplex immunochromatographic techniques, the localisation of several binding lines with different specific reactants was the best technical solution.

The parameters of the triple fluorescence immunochromatography system, such as artificial antigen scribing position, scribing concentration and GAM-IgG scribing concentration, were optimized by orthogonal test L9 (3)^3^ analysis. The results showed that the triple fluorescence immunochromatography system was optimized when the following conditions were met: SAs-BSA at T3 and 0.8 mg/ml (Table 1), FQs-BSA at T2 and 0.8 mg/ml (Table 2), TCs-BSA at T1 and 1.2 mg/ml (Table 3). The concentration of GAM-IgG was 1.5 mg/ml. The results obtained showed how the fluorescence intensity of each group of immunoreactants varies with their location. The fluorescence intensity of TCs-BSA, FQs-BSA and SAs-BSA reached its maximum when they were located at T1, T2 and T3, respectively.

**TABLE 1:** Orthogonal test to analyse the results of optimisation of L9 (3)^3^ on SAs-BSA concentration, GAM-IgG concentration and paddle position.

Number	T-line position	SAs-BSA concentration (mg/ml)	GAM-IgG concentration (mg/ml)	Fluorescence intensity (CPS)	T/C Value
1	T1	0.4	1.0	24,032	0.21
2	T1	0.6	1.5	25,763	0.23
3	T1	0.8	2.0	26,348	0.24
4	T2	0.4	1.5	10,320	0.18
5	T2	0.6	2.0	11,763	0.20
6	T2	0.8	1.0	12,538	0.35
7	T3	0.4	2.0	10,436	0.44
8	T3	0.6	1.0	11,873	0.47
9	T3	0.8	1.5	12,540	0.64

**TABLE 2:** Orthogonal test to analyse the results of optimisation of L9 (3)^3^ on FQs-BSA concentration, GAM-IgG concentration and paddle position.

Number	T-line position	FQs-BSA concentration (mg/ml)	GAM-IgG concentration (mg/ml)	Fluorescence intensity (CPS)	T/C Value
1	T1	0.8	1.0	13,654	0.47
2	T1	1.0	1.5	12,098	0.42
3	T1	1.2	2.0	11,012	0.21
4	T2	0.8	1.5	12,987	0.56
5	T2	1.0	2.0	12,653	0.22
6	T2	1.2	1.0	13,964	0.44
7	T3	0.8	2.0	8,740	0.23
8	T3	1.0	1.0	9,075	0.41
9	T3	1.2	1.5	10,237	0.48

**TABLE 3:** Orthogonal test to analyse the results of optimisation of L9 (3)^3^ on TCs-BSA concentration, GAM-IgG concentration and paddle position.

Number	T-line position	TCs-BSA concentration (mg/ml)	GAM-IgG concentration (mg/ml)	Fluorescence intensity (CPS)	T/C Value
1	T1	0.8	0.5	10,765	0.37
2	T1	1.0	1.0	11,796	0.51
3	T1	1.2	1.5	12,780	0.62
4	T2	0.8	1.0	8,653	0.45
5	T2	1.0	1.5	9,462	0.49
6	T2	1.2	0.5	10,723	0.58
7	T3	0.8	1.5	6,729	0.26
8	T3	1.0	0.5	7,862	0.49
9	T3	1.2	1.0	8,972	0.43

**FIGURE 4:**
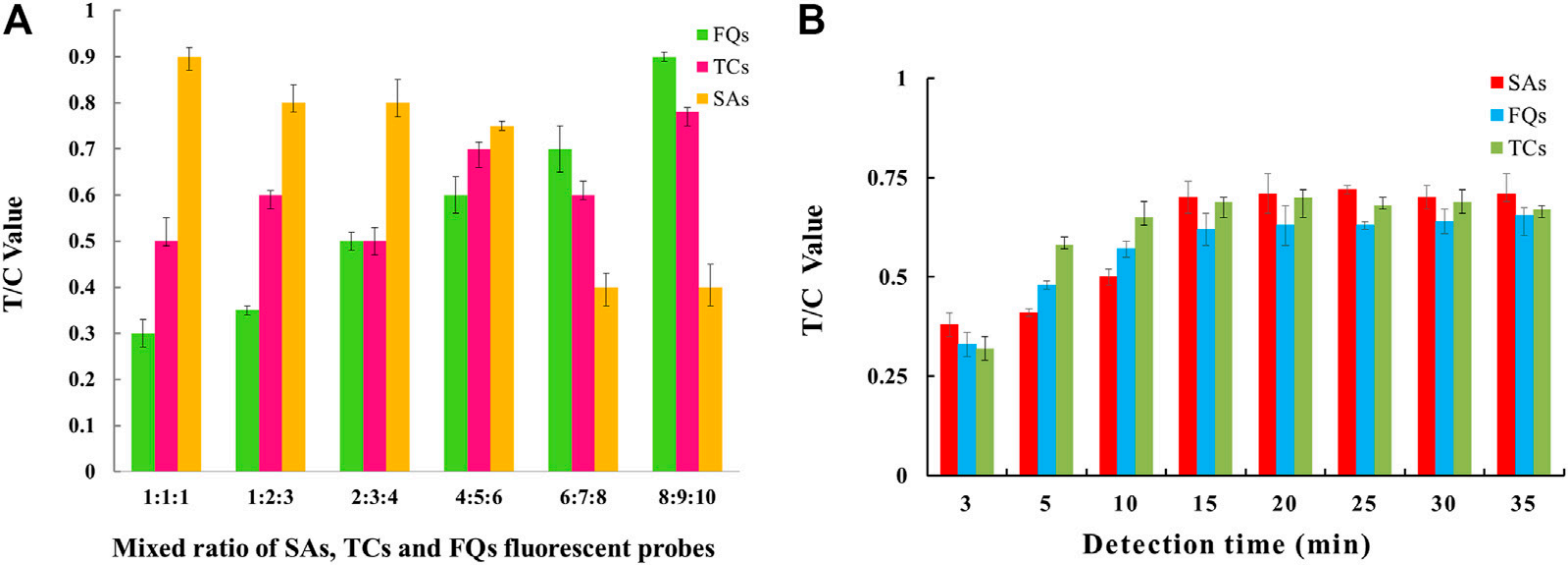
Parameter optimisation of three-multiple-EuNPs-FIA. **(A)** Effect of probe mixing ratio on the detection capability of three-multiple-EuNPs-FIA. **(B)** Effect of reaction time duration on the detection capacity of three-multiple-EuNPs-FIA.

The EuNPs-FIA was assembled according to the above conditions, and the parameters such as the time required for the reaction of their probe mixing ratios were optimized. The results showed that the detection capability of the three T-lines could be achieved at a mixing ratio of 6:5:4 for the three fluorescent probes of SAs, TCs and FQs (Figure 4A). The effect of reaction time on the analytical performance of the fluorescence immunochromatography system was that as the reaction time increases, the fluorescent probes achieve better binding to the antigen and the fluorescence intensity of the C and T lines gradually increases, reaching a peak at 15 min and remaining stable, so 15 min was selected as the optimal immunoreaction time for the system (Figure 4B).

### Specificity and Sensitivity Assessment

**FIGURE 5:**
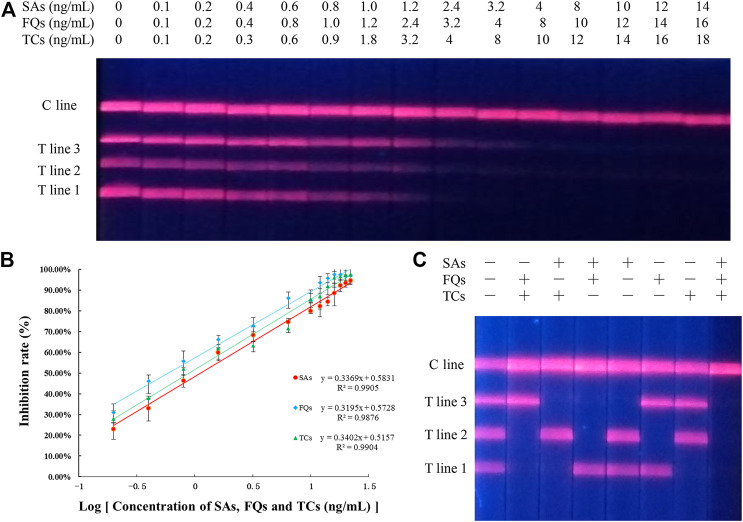
Sensitivity and specificity analysis of three-multiple-EuNPs-FIA. **(A)** Simultaneous sensitivity analysis of SAs, FQs and TCs by three-multiple-EuNPs-FIA. **(B)** The standard curve for SAs, FQs and TCs by three-multiple-EuNPs-FIA. **(C)** Specific analysis of three-multiple-EuNPs-FIA.

The EuNPs-FIA was formally established under optimised experimental conditions, by separately testing a series of antibiotic standard solutions at different concentrations, it was observed that the fluorescence intensity on the T-line gradually diminished until it disappeared as the concentration of the standard increased, thus obtaining a cut-off value for the monitoring system. When using EuNPs-FIA under the same conditions, the T-line fluorescence intensity disappears at standard concentrations of 4.0, 3.2 and 2.4 ng/ml and allows for simultaneous detection of all three antibiotics (Figure 5A). Based on the cut-off values, a standard curve was created by reading the test strips using an EuNPs fluorescent strip reader thereby creating a standard curve (Figure 5B), expressing the relationship between inhibition and the logarithm of the antibiotic concentration and calculating the cross-reactivity (CR) between each antibiotic (Supplementary Table S1–S3). The high cross-reactivity between antibiotics of the same class, due to the same parent nucleus structure, offered the possibility of simultaneous detection of multiple targets to be tested.

The specificity of the method was assessed by calculating the cross-reactivity between 13 types of SAs, four types of TCs and seven types of FQs separately, and the results showed that there was a significant cross-reactivity of the screened SAs-mAb for 13 types of SAs, a significant cross-reactivity of the screened TCs-mAb for four types of TCs and a significant cross-reactivity of the screened FQs-mAb for seven types of FQs cross-reactivity. The three monoclonal antibodies were shown to be group-specific monoclonal antibody and the EuNPs-FIA allowed the simultaneous detection of 24 structurally different antibiotic residues (Supplementary Figure S5). And there was no cross-reactivity between the three families of antibiotics (Figure 5C), indicating that the three classes of antibiotics do not affect each other. The class specificity of the three monoclonal antibodies was excellent.

### Spiked Recovery Tests and Actual Sample Analysis

**FIGURE 6:**
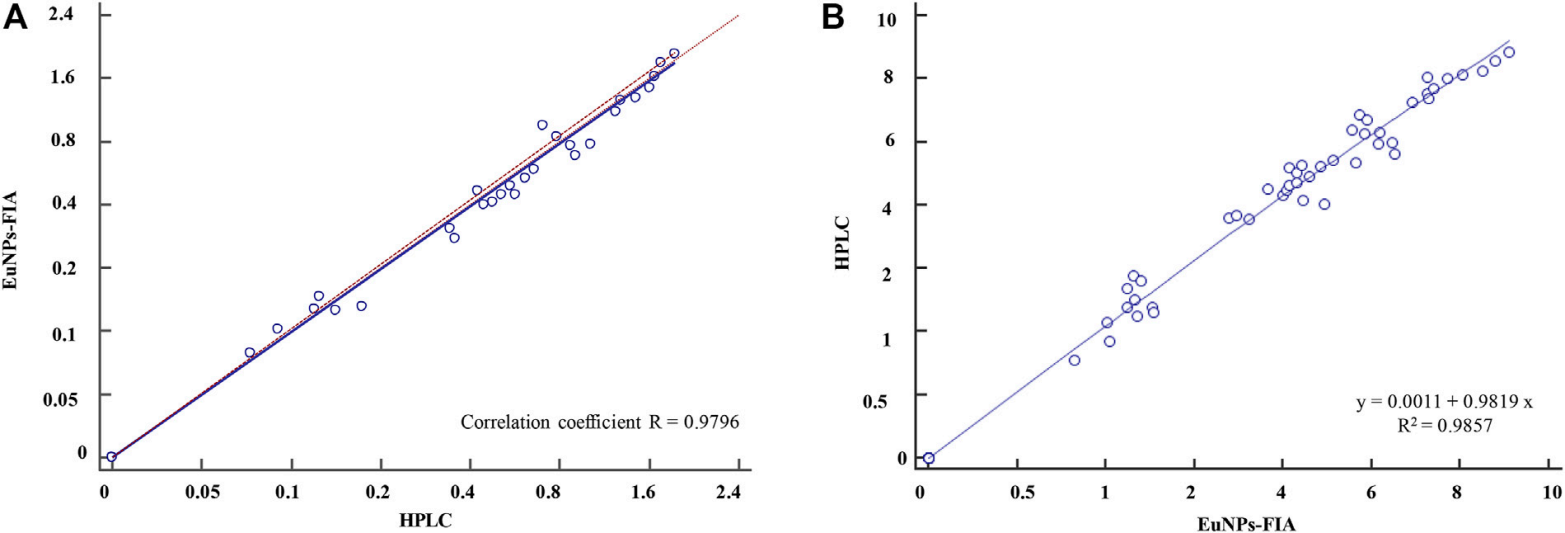
Comparison of EuNPs-FIA and HPLC. **(A)** Comparative method scatter diagram of EuNPs-FIA and HPLC. **(B)** Passing-Bablok regression.

The accuracy and precision of the method was evaluated by spiked recovery experiments. Different concentrations of analytes were added to HPLC-validated negative blank milk samples and then assayed by EuNPs-FIA. The results showed (Table 4) that the mean recoveries of the three types of mixed reference analytes ranged from 92.1 to 106.2% at spiked concentrations of 0.1–2.4 ng/ml with a relative standard deviation of less than 8.75%. The assay results were validated by using HPLC with correlation coefficients above 0.97 (Figure 6A).

**TABLE 4:** Recovery rate determination of SAs, TCs and FQs in milk sample from three-multiple-EuNPs-FIA analysis.

Analyte	Spiked level (ng/ml)	Intra-assay		
		Detected amount (ng/ml)	Recovery rate	RSD (*n* = 3)
SMD	0.1	0.09	92.10%	4.16%
	0.2	0.18	105.00%	8.03%
	0.4	0.45	106.20%	7.76%
	0.6	0.61	100.56%	6.30%
	0.8	0.83	102.08%	7.37%
	1.0	1.01	103.33%	4.88%
	1.2	1.21	101.94%	5.67%
	2.4	2.34	98.19%	2.41%
TC	0.1	0.12	106.1%	3.11%
	0.2	0.19	103.33%	7.34%
	0.4	0.44	103.33%	6.80%
	0.6	0.63	105.00%	2.33%
	0.8	0.82	102.98%	3.17%
	1.0	1.1	105.67%	6.09%
	1.2	1.23	101.11%	6.30%
	2.4	2.39	100.83%	3.08%
ENR	0.1	0.12	103.33%	8.10%
	0.2	0.22	103.33%	8.75%
	0.4	0.45	105.00%	4.86%
	0.6	0.65	103.33%	3.23%
	0.8	0.87	99.58%	5.82%
	1.0	1.01	97.33%	3.31%
	1.2	1.13	97.78%	7.34%
	2.4	2.35	99.31%	2.93%

To further validate the established method, 60 different samples purchased from the whole province of Zhejiang were determined using EuNPs-FIA and HPLC. The results demonstrated a satisfactory agreement between the detection values of EuNPs-FIA and HPLC (*R* ^2^ > 0.98) (Figure 6B). This indicated that the EuNPs-FIA can be used for the analysis of real samples (Table 5). Therefore, it was effective for controlling the potential risk of antibiotic residues in food and could be used for direct dilution testing of milk samples with a detection time of 15 min, no loss of detection sensitivity and almost complete recovery of the added target analytes.

**TABLE 5:** Detection of SAs, TCs and FQs in egg, milk, chicken and honey samples by three-multiple-EuNPs-FIA and HPLC.

Sample	EuNPs-FIA (μg/kg)			HPLC (μg/kg)		
	SAs	TCs	FQs	SAs	TCs	FQs
honey 01	—	3.06 ± 0.24	—	—	2.82 ± 0.02	—
honey 02	8.15 ± 0.13	—	—	8.82 ± 0.31	—	—
honey 03	—	—	—	—	—	—
honey 04	—	—	5.68 ± 0.23	—	—	6.58 ± 0.07
honey 05	—	—	—	—	—	—
honey 06	7.20 ± 0.34	—	—	7.99 ± 0.19	—	—
honey 07	—	—	—	—	—	—
honey 08	8.68 ± 0.29	—	—	8.54 ± 0.17	—	—
honey 09	—	—	—	—	—	—
honey 10	—	—	—	—	—	—
honey 11	—	—	—	—	—	—
honey 12	—	5.04 ± 0.29	—	—	4.35 ± 0.03	—
honey 13	6.14 ± 0.27	—	3.08 ± 0.14	7.52 ± 0.31	—	4.66 ± 0.05
honey 14	—	—	—	—	—	—
honey 15	—	—	—	—	—	—
honey 16	—	4.35 ± 0.27	—	—	5.62 ± 0.26	—
honey 17	—	—	—	—	—	—
honey 18	—	—	7.02 ± 0.23	—	—	8.08 ± 0.17

Sample	EuNPs-FIA (μg/kg)			HPLC (μg/kg)		
	SAs	TCs	FQs	SAs	TCs	FQs
chicken 01	—	—	4.54 ± 0.22	—	—	4.66 ± 0.04
chicken 02	—	—	—	—	—	—
chicken 03	—	—	4.10 ± 0.16	—	—	5.32 ± 0.12
chicken 04	2.23 ± 0.24	—	—	1.15 ± 0.11	—	—
chicken 05	3.73 ± 0.19	—	5.20 ± 0.17	4.03 ± 0.26	—	6.25 ± 0.18
chicken 06	—	—	—	—	—	—
chicken 07	—	2.53 ± 0.24	—	—	2.93 ± 0.17	—
chicken 08	—	—	—	—	—	—
chicken 09	—	—	—	—	—	—
chicken 10	—	—	—	—	—	—
chicken 11	—	—	—	—	—	—
chicken 12	—	—	6.69 ± 0.29	—	—	5.72 ± 0.19

Sample	EuNPs-FIA (μg/L)			HPLC (μg/L)		
	SAs	TCs	FQs	SAs	TCs	FQs
milk 01	—	—	—	—	—	—
milk 02	—	—	—	—	—	—
milk 03	6.05 ± 0.25	4.85 ± 0.24	—	5.90 ± 0.13	5.65 ± 0.12	—
milk 04	—	—	—	—	—	—
milk 05	—	—	—	—	—	—
milk 06	—	—	19.4 ± 0.07	—	—	20.6 ± 0.15
milk 07	—	—	—	—	—	—
milk 08	—	—	27.9 ± 0.21	—	—	30.9 ± 0.13
milk 09	—	—	—	—	—	—
milk 10	—	26.7 ± 0.29	—	—	21.6 ± 0.14	—
milk 11	—	—	—	—	—	—
milk 12	—	—	—	—	—	—
milk 13	6.4 ± 0.31		17.9 ± 0.15	8.32 ± 0.09	—	18.63 ± 0.02
milk 14	—	—	—	—	—	—
milk 15	—	—	—	—	—	—
milk 16	28.8 ± 0.21	—	—	31.4 ± 0.15	—	—
milk 17	—	—	—	—	—	—
milk 18	—	—	—	—	—	—
milk 19	—	4.25 ± 0.22	—	—	4.85 ± 0.02	—
milk 20	—	—	—	—	—	—

Sample	EuNPs-FIA (μg/kg)			HPLC (μg/kg)		
	SAs	TCs	FQs	SAs	TCs	FQs
egg 01	—	—	—	—	//	—
egg 02	—	—	—	—	—	—
egg 03	—	—	2.01 ± 0.22	—	—	2.35 ± 0.03
egg 04	—	—	—		—	—
egg 05	13.51 ± 0.24	—	—	15.30 ± 0.24	—	—
egg 06	—	—	—	—	—	—
egg 07	—	—	—	—	—	—
egg 08	—	—	—	—	—	—
egg 09	—	17.6 ± 0.19	—	—	14.13 ± 0.19	—
egg 10	—	—	6.39 ± 0.14	—	—	8.17 ± 0.17

## Conclusion

In this study, artificial antigens were prepared by designing and synthesizing the parent nucleus structures of each of SAs, TCs and FQs, coupling the parent nucleus structure antigens of FQs, SAs and TCs with carrier proteins by carbodiimide and N-hydroxysuccinimide-activated ester methods, respectively, and immunising mice to obtain herd immune monoclonal antibodies. The EuNPs-FIA detection system was constructed by combining the novel fluorescent nanomaterial europium, immunolateral flow test paper and monoclonal antibodies. It not only resolved the problem of low sensitivity of traditional detection methods, but also enabled the detection of almost an entire class of antibiotic drug residues by a single test line. The combination of the three T-lines was capable of detecting four types of TCs, seven types of FQs and 13 types of SAs simultaneously, covering 24 antibiotic residues in different structural forms. The number of compounds that can be detected and controlled in food safety and ecological monitoring has been increased, enabling public environmental safety to be ensured. The detection results were highly correlated with those of HPLC. The method therefore exhibits high sensitivity, good accuracy and reliability and satisfactory selectivity. It could contribute to the detection and monitoring of sulphonamide, tetracycline and fluoroquinolone antibiotic residues in food and to the monitoring of the quality of the environment in which people live. Currently, we are investigating the use of a population immunogenic monoclonal antibody on test strips to recognise more different classes of target to be tested, enabling the detection of multiple classes of targets in a single T-line. We hope that this multiplex test strip analysis will be useful in maintaining food safety.

## Data Availability

The raw data supporting the conclusion of this article will be made available by the authors, without undue reservation.
