# Supplementary material for: Europium Fluorescent Nanoparticles-Based Multiplex Lateral Flow Immunoassay for Simultaneous Detection of Three Antibiotic Families Residue
Source: Front Chem. 2021 Dec 20;9:793355. doi: 10.3389/fchem.2021.793355 (PMC8722402; doi:10.3389/fchem.2021.793355)
Supplement: Supplementary file 1 [file DataSheet1.doc]

**S1. Preparation and identification of FQs-artificial antigens.**

**
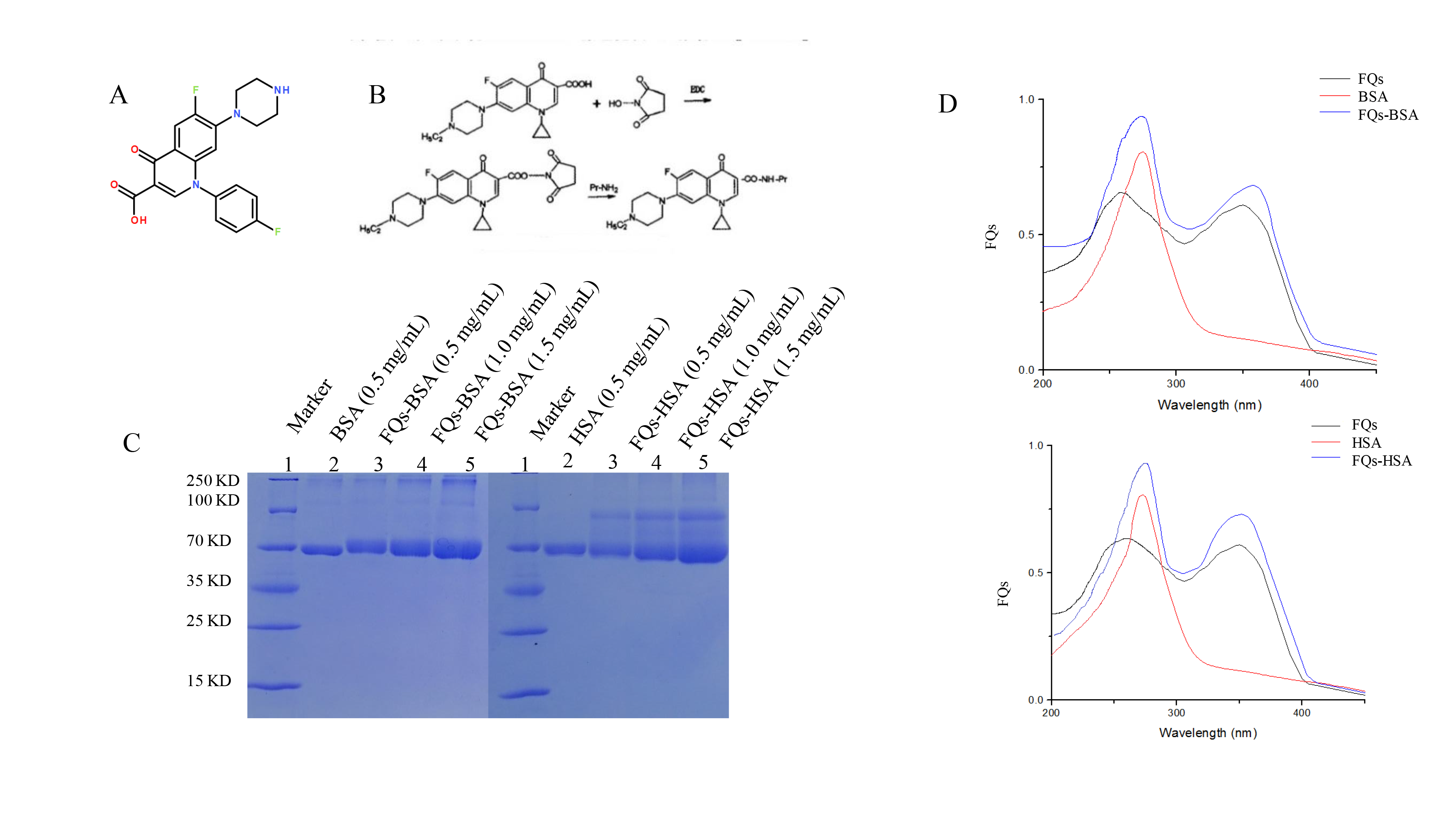
**

Fig.S1 Preparation and identification of FQs-artificial antigens. (A) The parent nucleus structure of FQs. (B) Synthetic route to FQs-artificial antigens. (C) SDS-PAGE analysis of FQs-BSA and FQs-HSA. (D) UV-Vis spectroscopic identification results of FQs-artificial antigens.

Fig.S1C showed that FQs-BSA band was found after bovine serum albumin band appeared, reflecting obvious tailing phenomenon. Therefore, FQs successfully combined with the residue of BSA to form a conjugate. Significant hysteresis indicated that more FQs was attached to BSA. In addition, FQs-HSA band also reflects this feature, indicating that FQs has been successfully coupled to HSA.

**S2. Preparation and identification of SAs-artificial antigens.**

**
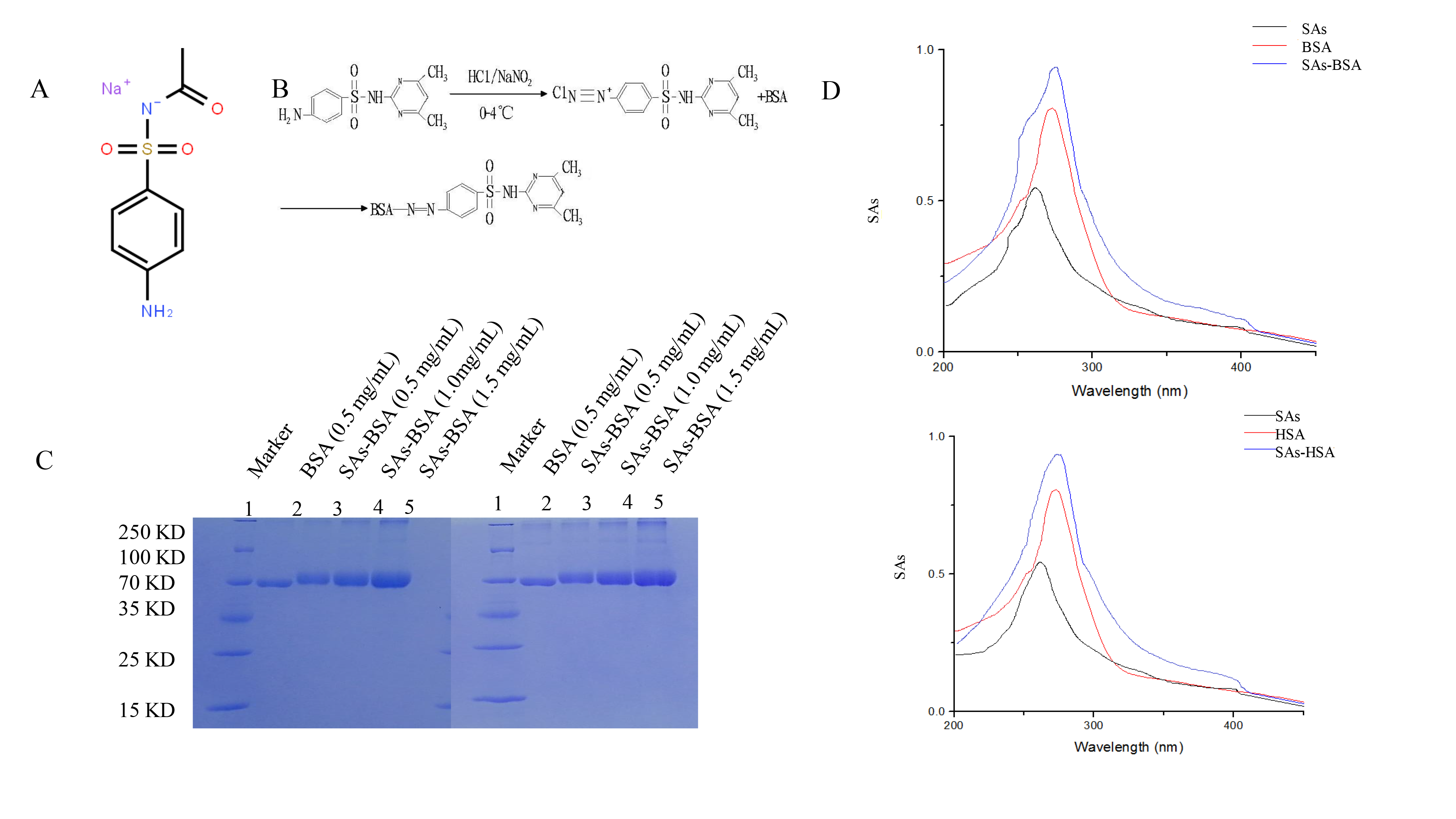
**

Fig.S2 Preparation and identification of SAs-artificial antigens. (A) The parent nucleus structure of SAs. (B) Synthetic route to SAs-artificial antigens. (C) SDS-PAGE analysis of SAs-BSA and SAs-HSA. (D) UV-Vis spectroscopic identification results of SAs-artificial antigens.

Fig.S2C showed that SAs-BSA band was found after bovine serum albumin band appeared, reflecting obvious tailing phenomenon. Therefore, SAs successfully combined with the residue of BSA to form a conjugate. Significant hysteresis indicated that more SAs was attached to BSA. In addition, SAs-HSA band also reflects this feature, indicating that SAs has been successfully coupled to HSA.

**S3. Preparation and identification of TCs-artificial antigens.**


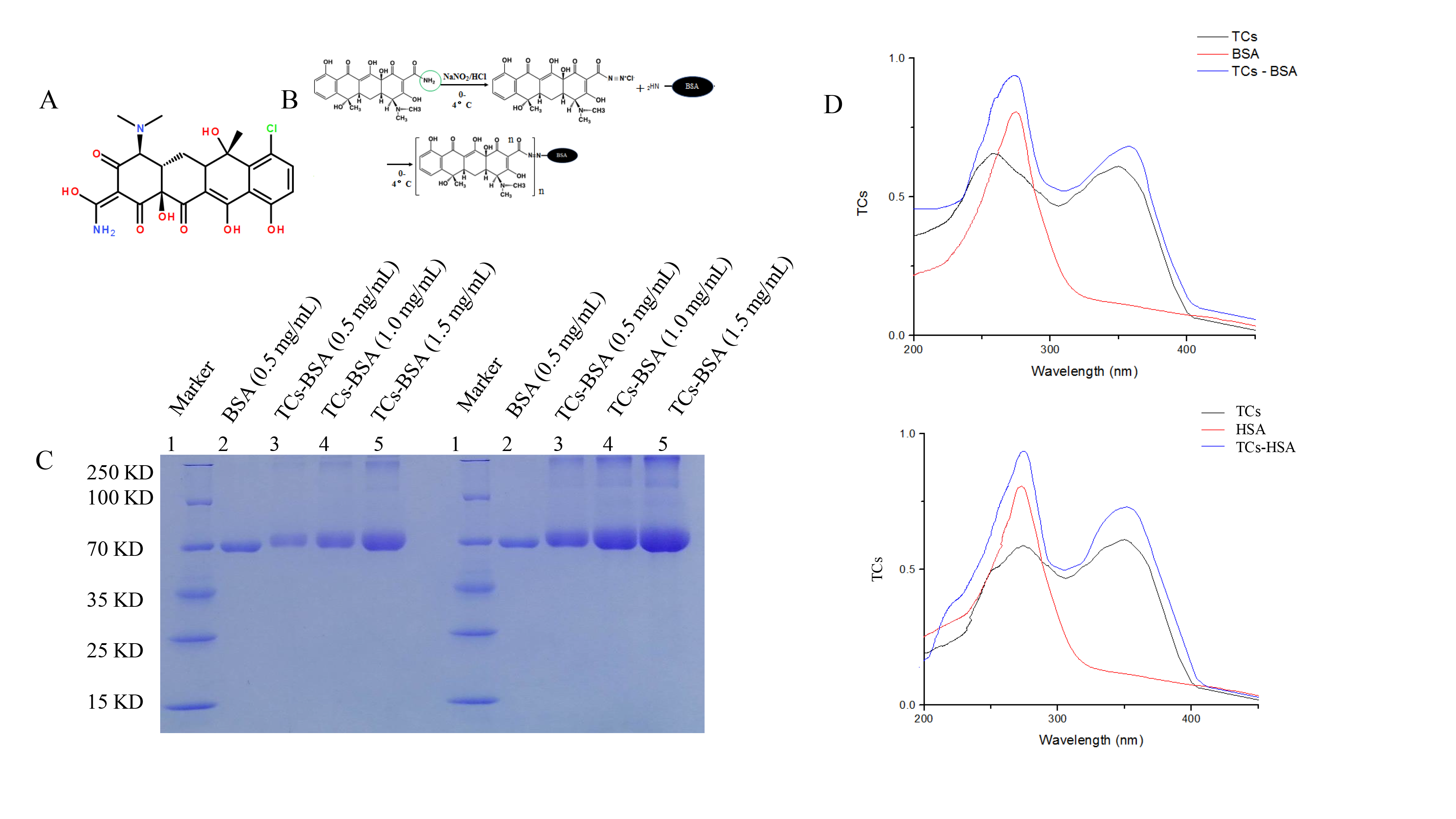


Fig.S3 Preparation and identification of TCs-artificial antigens. (A) The parent nucleus structure of TCs. (B) Synthetic route to TCs-artificial antigens. (C) SDS-PAGE analysis of TCs-BSA and TCs-HSA. (D) UV-Vis spectroscopic identification results of TCs-artificial antigens.

Fig.S3C showed that TCs-BSA band was found after bovine serum albumin band appeared, reflecting obvious tailing phenomenon. Therefore, TCs successfully combined with the residue of BSA to form a conjugate. Significant hysteresis indicated that more TCs was attached to BSA. In addition, TCs-HSA band also reflects this feature, indicating that TCs has been successfully coupled to HSA.

**S4. Identification of Monoclonal Antibody.**

After optimization by checkerboard method, indirect competitive enzyme-linked immunosorbent assay was used to detect antiserum and identify antibodies. The specific operation is as follows. The 96-well plate coated with FQs-BSA, SAs-BSA and TCs-BSA were sealed at 4 ℃ for 16 hours respectively. After the reaction, the 96-well plate was dried at 37 ℃ for 4 hours and stored at 4 ℃ for later use. Add PBS and diluted ENR, SMZ and TC standard at 50 μL/well to the closed 96-well plate respectively. Then, the anti-FQs, anti-SAs and anti-TCs monoclonal antibody and goat anti-mouse horseradish peroxidase were diluted respectively, and 50 μL/well was added to the 96-well plate. React for 30 minutes at 25 ℃. After the reaction was completed, the plate was washed three times with PBST. Then, TMB developer was added in an amount of 100 μL/well, and the reaction was carried out at 25 ℃ for 15 minutes in the dark. Add the stop solution in an amount of 50 μL/well, and measure the OD value of each well at 450 nm using a microplate reader.

**
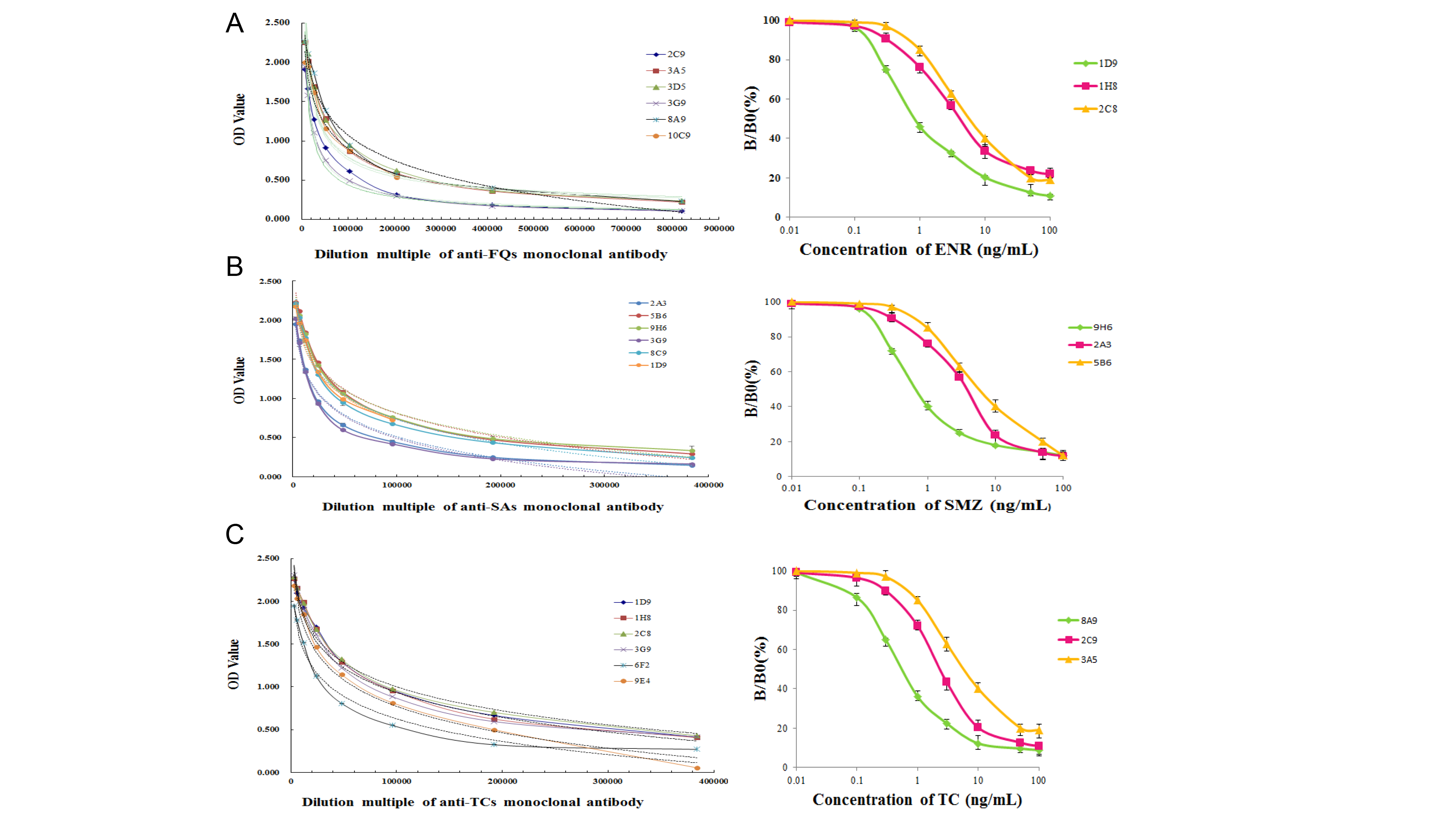
**

Fig.S4 (A) Valence identification of different FQs monoclonal antibodies. (B) Valence identification of different SAs monoclonal antibodies. (C) Valence identification of different TCs monoclonal antibodies.

The monoclonal antibodies with the highest titres, 8A9, 9H6 and 1D9, were selected for subsequent testing based on the calculated results.

**S5. Single-EuNPs-FIA specificity analysis.**


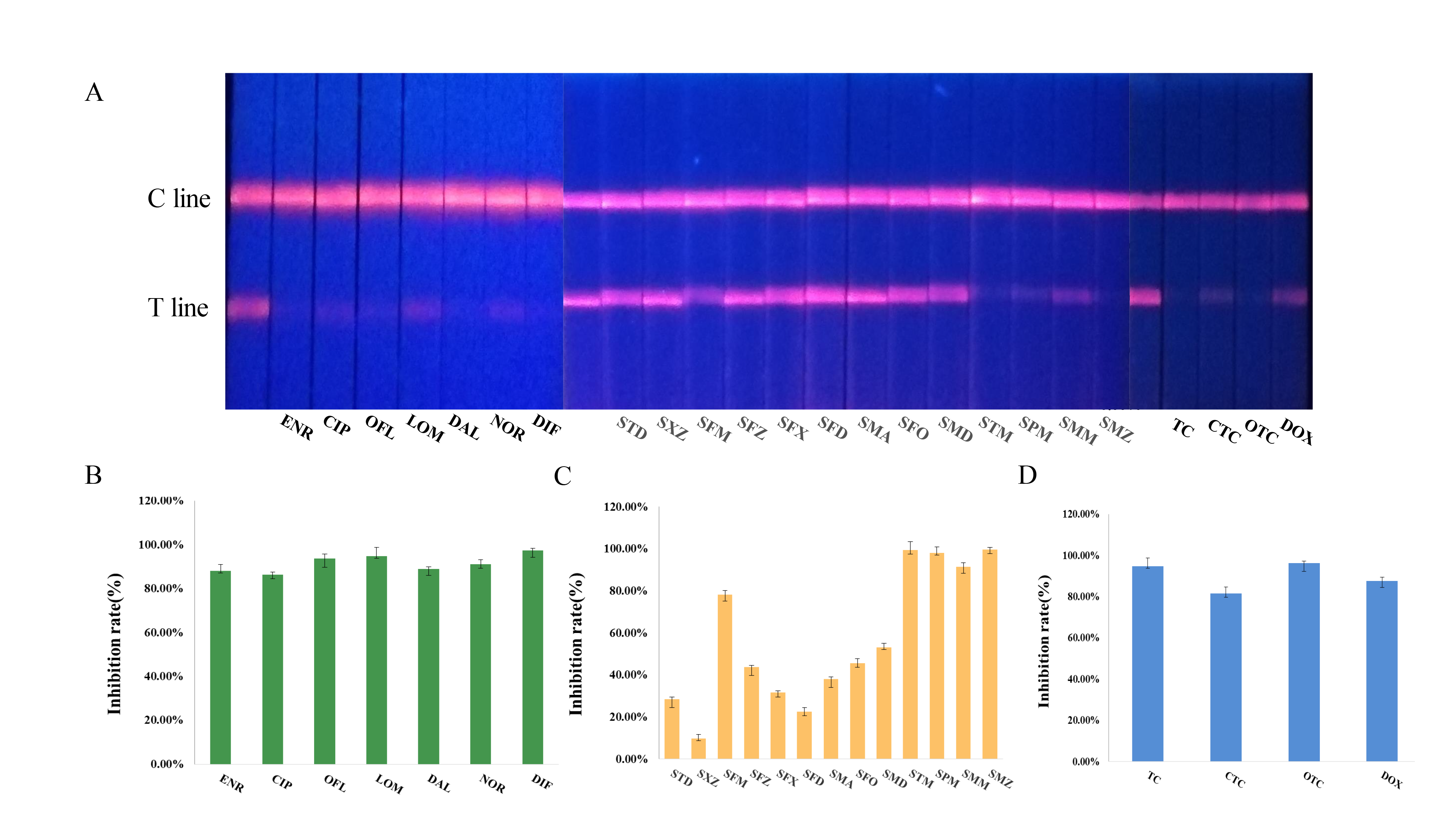


Fig.S5 Single-EuNPs-FIA specificity analysis. (A)Specific analysis of single-EuNPs-FIA. (B) Analysis of the specificity of 7 types of FQs (C) Analysis of the specificity of 13 types of SAs (D) Analysis of the specificity of 4 types of TCs

To determine the cross-reactivity of this anti-SAs, anti-TCs and anti-FQs mAb, indirect competitive ELISA was carried out with antibiotics of the same family and other antibiotics including STD, SXZ, SFM, SFZ, SFX, SFD, SMA, SFO, SMD, STM, SPM, SMM, SMZ, TC, CTC, OTC, DOX, ENR, CIP, OFL, LOM, DAL, NOR and DIF. The result indicated that this anti-SAs, anti-TCs and anti-FQs mAb had good population immune specificity and cross-reacted to varying degrees with other antibiotics in its class. (Table S1-S3).

**Table S1.** Cross-reactivities of anti-SAs mAb with antibiotics of the same family.

| antibiotics | Cross-Reactivity (%) |
| --- | --- |
| STD | 28.9 |
| SXZ | 21.11 |
| SFM | 77.17 |
| SFZ | 25.47 |
| SFX | 32.71 |
| SFD | 28.49 |
| SMA | 23.06 |
| SFO | 47.33 |
| SMD | 54.97 |
| STM | 99.80 |
| SPM | 95.84 |
| SMM | 88.68 |
| SMZ | 100.00 |

**Table S2.** Cross-reactivities of anti-TCs mAb with antibiotics of the same family.

| antibiotics | Cross-Reactivity (%) |
| --- | --- |
| TC | 100.00 |
| CTC | 66.68 |
| OTC | 98.81 |
| DOX | 84.89 |

**Table S3.** Cross-reactivities of anti-FQs mAb with antibiotics of the same family.

| antibiotics | Cross-Reactivity (%) |
| --- | --- |
| ENR | 88.82 |
| CIP | 96.28 |
| OFL | 91.35 |
| LOM  DAL  NOR  DIF | 97.36  93.71  90.42  100.00 |

**Table S4.** Comparison of the analytical performance of different detection probes for the detection of antibiotic residues.

| Detection probes | Analytes | Sample | Detection  time (min) | LOD | References |
| --- | --- | --- | --- | --- | --- |
| AuNPs | sulfadiazine, sulfamerazine, sulfadimethoxine, sulfamonomethoxie, | honey | 25 | 10 ng/mL  5 ng/mL  10 ng/mL  25 ng/mL | Chen et al., 2017 |
| AuNPs | clindamycin  lincomycin  pirimycin | - | - | 1 ng/mL  10 ng/mL  25 ng/mL | Guo et al., 2020 |
| AuNPs | tylenol | milk | - | 17.7 ng/mL | Huang et al., 2019 |
| EuNPs | streptomycin | milk | 20 | 0.58 ng/mL | Wang et al., 2020 |
| QDs-IFA | ofloxacin chloramphenicol  streptomycin | milk | 20 | 0.3 ng/mL  0.12 ng/mL  0.2 ng/mL | Taranova et al., 2015 |
| UCNP-LFIC | Tetracycline  oxytetracycline  chlortetracycline  doxycycline | - | 10 | 0.032 ng/mL  0.045 ng/mL  0.029 ng/mL  0.022 ng/mL | Xu et al., 2020 |
| EuNPs-FIA | SAs  TCs  FQs | honey, milk,egg and chicken | 15 | 0.015 ng/mL  0.023 ng/mL  0.031 ng/mL | This study |

AuNPs: gold nanoparticles

EuNPs: europium nanoparticles

QDs-IFA: quantum dots immunofluorescence assay;

UCNP-LFIC: up-conversion nanoparticles lateral-flow immunochromatographic.

EuNPs-FIA: europium nanoparticle-based fluorescent immunoassay

The limit of detection (LOD) was calculated as the mean of the measured content of blank different samples (n = 20) plus three standard deviations (mean + 3SD) .We have calculated the LODs of SAs, TCs and FQs according to this calculation method were 0.015, 0.023 and 0.031 ng/mL, respectively.

**References**

Chen, Y. N., Liu, L. Q., Xu, L.G., and Xu, C.L. (2017). Gold immunochromatographic sensor for the rapid detection of twenty-six sulfonamides in foods. Nano Research 8, 2833-2844. doi:10.1007/s12274-017-1490-x.

Guo, L. L., Wu, X. L., Liu, L. Q., and Xu, C. L. (2020). Gold Immunochromatographic Assay for Rapid On-Site Detection of Lincosamide Residues in Milk, Egg, Beef, and Honey Samples. Biotechnology journal 1, 1900174. doi:10.1002/biot.201900174.

Huang, J. X., Yao, C. Y., Yang, J. Y., Li, Z. F., and He, F. (2019). Design of Novel Haptens and Development of Monoclonal Antibody-Based Immunoassays for the Simultaneous Detection of Tylosin and Tilmicosin in Milk and Water Samples. Biomolecules 9, 770. doi:10.3390/biom9120770

Taranova, N.A., Berlina, A.N., Zherdev, A.V., and Dzantiev, B.B. (2015). ‘Traffic light’ immunochromatographic test based on multicolor quantum dots for the simultaneous detection of several antibiotics in milk. Biosensors and Bioelectronics 63, 255-261. doi:10.1016/j.bios.2014.07.049

Wang, Z. L, Sun, Y. Z., Liang, D. M., Zeng, Y. Y., He, S ., Mari, G. M., and Jiang, H. Y. (2020). Highly sensitive chromatographic time-resolved fluoroimmunoassay for rapid onsite detection of streptomycin in milk. Journal of dairy science 10, 8750-8760. doi:10.3168/JDS.2020-18393.

Xu, Y., Ma, B., Chen, E. J., Yu, X.P., Sun, C. X., and Zhang, M. Z. (2020) Functional Up-Conversion Nanoparticle-Based Immunochromatography Assay for Simultaneous and Sensitive Detection of Residues of Four Tetracycline Antibiotics in Milk. Frontiers in Chemistry 8, 759. [doi:0.3389/fchem.2020.00759](https://doi.org/10.3389/fchem.2020.00759)
